# Characterising atypical *Candida albicans* clinical isolates from six third-level hospitals in Bogotá, Colombia

**DOI:** 10.1186/s12866-015-0535-0

**Published:** 2015-10-05

**Authors:** Giovanni Rodríguez-Leguizamón, Alessandro Fiori, Luisa F. López, Beatriz L. Gómez, Claudia M. Parra-Giraldo, Arley Gómez-López, Carlos F. Suárez, Andrés Ceballos, Patrick Van Dijck, Manuel A. Patarroyo

**Affiliations:** School of Medicine and Health Sciences, Universidad del Rosario, Bogotá, Colombia; VIB Department of Molecular Microbiology, Leuven, Belgium; KU Leuven Laboratory of Molecular Cell Biology, Leuven, Belgium; Medical and Experimental Mycology Unit, Corporación para las Investigaciones Biológicas (CIB), Medellín, Colombia; Infectious Diseases Research Group, Microbiology Department, Pontificia Universidad Javeriana, Bogotá, Colombia; Molecular Biology and Immunology Department, Fundación Instituto de Inmunología de Colombia (FIDIC), Bogotá, Colombia; Biomathematics Department, Fundación Instituto de Inmunología de Colombia (FIDIC), Bogotá, Colombia

**Keywords:** *Candida albicans*/*Candida africana*, Atypical *Candida albicans*, MALDI TOF-MS, Chlamydospores

## Abstract

**Background:**

*Candida* species are the most frequently found fungal pathogens causing nosocomial disease in a hospital setting. Such species must be correctly identified to ensure that appropriate control measures are taken and that suitable treatment is given for each species. *Candida albicans* is causing most fungal disease burden worldwide; the challenge lies in differentiating it from emerging atypical, minor and related species such as *Candida dubliniensis* and *Candida africana.* The purpose of this study was to compare identification based on MALDI-TOF MS to standard identification systems using a set of nosocomial isolates.

**Methods:**

Eleven nosocomial samples were collected from 6 third-level hospitals in Bogotá, Colombia. All the samples were identified by combining MALDI-TOF MS with morphological characters, carbohydrate assimilation and molecular markers (D1/D2 and *HWP1*).

**Results:**

The present work describes the first collection of atypical Colombian *Candida* clinical isolates; these were identified as *Candida albicans/Candida africana* by their MALDI-TOF MS profile. Phenotypical characteristics showed that they were unable to produce chlamydospores, assimilate trehalose, glucosamine, N- acetyl-glucosamine and barely grew at 42 °C, as would be expected for *Candida africana*. The molecular identification of the D1/D2 region of large subunit ribosomal RNA and *HWP1* hyphal cell wall protein 1 sequences from these isolates was consistent with those for *Candida albicans*. The mass spectra obtained by MALDI-TOF MS were analysed by multi-dimensional scaling (MDS) and cluster analysis, differences being revealed between *Candida albicans*, *Candida africana*, *Candida dubliniensis* reference spectra and two clinical isolate groups which clustered according to the clinical setting, one of them being clearly related to *C. albicans*.

**Conclusion:**

This study highlights the importance of using MALDI-TOF MS in combination with morphology, substrate assimilation and molecular markers for characterising *Candida albicans*-related and atypical *C. albicans* species, thereby overcoming conventional identification methods. This is the first report of hospital-obtained isolates of this type in Colombia; the approach followed might be useful for gathering knowledge regarding local epidemiology which could, in turn, have an impact on clinical management. The findings highlight the complexity of distinguishing between typical and atypical *Candida albicans* isolates in hospitals.

## Background

*Candida albicans* is the pathogenic fungus most commonly found in the general population; its ease of transmission in hospitals causes high comorbidity rates [1, 2]. This species causes mucosal infections, mainly oropharyngeal candidiasis (OPC) and vulvovaginal candidiasis (VVC); however, the relevant nosocomial presentation is related to candidaemia and invasive candidiasis which is associated with high mortality rates (~49 %) in an ICU [3]. *C. albicans* minor and related species have been identified worldwide and, interestingly, types may be associated with particular patterns of clinical presentation [4–8]. *C. dubliniensis* was described in 1995 as being the first *C. albicans*-related species, initially in patients having a background of HIV and infection in the oral cavity [4, 9]. *C. africana* was described in 2001 as being another related species associated with vaginal infection; one case of bloodstream infection has been reported in Chile [4, 5, 7].

The timely identification of a microorganism is one of the challenges for clinical practice and controlling hospital-acquired infection, thereby facilitating decision-making for therapeutic prescription and epidemiological surveillance. The identification methods currently available in hospitals may not be suitable for the precise identification of *C. albicans*-related species. A lack of chlamydospore formation, an inability to assimilate trehalose or amino sugars and poor growth at 42 °C are the most useful traits for distinguishing *C. africana* from *C. albicans* and *C. dubliniensis* [5, 6, 10, 11].

Molecular markers such as large-subunit rRNA D1/D2 domain and hyphal cell wall protein 1 (*HWP1*) DNA sequence are also useful tools for species identification [6, 10, 12]. Matrix-assisted laser desorption/ionisation time of flight mass spectrometry (MALDI-TOF MS) currently provides an accurate, rapid and relatively inexpensive approach to yeast typing, having ~97.6 % accuracy in identifying *Candida* species [13–18].

Nowadays, there are new challenges in detection-related medical mycology, identification and classification of pathogenic fungi. Regarding *C. albicans*, there is concern due to the identification of minor species such as *C. dubliniensis* and *C. albicans*-related ones (such as *C. africana* and *C. stellatoidea*) which have been grouped within the *C. albicans* complex [19]. These minor and bio-variant species cause disease at a specific anatomical location or site depending on the infecting species; precise identification by conventional methods has proven difficult to date [15, 19] and this situation could lead to overestimating the epidemiological prevalence of *C. albicans*. Misdiagnosis may mask such atypical organisms’ clinical implications related to their pathology and virulence [19, 20].

*Candida africana* seems to be a new player in the clinical picture of infectious diseases. Most reported infection attributed to this species is located in the genital tract; however, studies on this matter have been limited to date. Despite *C. africana* having been considered less pathogenic than *C. albicans* due to its anatomical localisation, the pathogenic consequences when infections occur at other sites of the host require further study [19, 21].

Taxonomic discussion concerning this new variant includes the fact that studies have demonstrated that *C. albicans* and *C. africana* are very close, as assessed by phylogenetic position based on studies of sequences from the D1/D2 region of their 28S ribosomal RNA genes. The same situation has occurred with internal transcribed spacer (ITS) sequence regions having 99.3–99.8 % identity, this being almost identical to *C. albicans* intraspecies values (99.8–100 %). Whether *C. africana* should be considered a new species or biovar is still open to discussion; however, some authors have considered that there are specific molecular markers such as the *HWP1* gene and additional different phenotypic traits such as carbon assimilation and no chlamydospore formation can establish a real difference from its closest relative *C. albicans* [22].

Our research group has thus been interested in evaluating how combining MALDI-TOF MS with morphology, substrate assimilation and molecular markers can be used to characterise these atypical *C. albicans* isolates from clinical settings. This initiative began after peculiar phenotypical traits were found in some clinical isolates taken from patients from six local hospitals diagnosed as having nosocomial infection by *C. albicans* (i.e., low cellular adhesion, smaller yeast size and slower growth).

## Methods

### Ethics statement

Committees from the following institutions approved this study: Universidad del Rosario’s ethics committee, covering its associated institutions (Hospital de Kennedy, Hospital Simón Bolívar, Hospital El Tunal, Hospital la Samaritana and Clínica de Occidente) and the Hospital San Ignacio’s ethics committee. Samples were taken as part of standard care and were irreversibly anonymised; following ethical guidelines, no patient consent was thus required for research purposes, taking into account that this is no longer considered personal data.

### Strains and isolates

Isolates were collected from two sources; 40 nosocomial isolates were obtained from 10 third-level hospitals and then identified by MALDI-TOF MS in a first batch. The second phase involved 240 isolates processed by MALDI-TOF MS from hospital samples from the San Ignacio HUSI teaching hospital in Bogotá, Colombia. Table 1 gives such isolates’ clinical characteristics, referring to the origin of the samples and relevant clinical data.

**Table 1:** Clinical information regarding the isolates studied

Isolate	Patient gender	Age (in years)	Source	AF treatment	Clinical outcome	Clinical setting	Locality
CO_R6	F	55	Urine	None	Dead	ICU	Kennedy
CO_R41	M	1 month	Blood	Amphotericin B	Alive	ICU	Usaquen
CO_R111	M	18	Urine	Fluconazole	Alive	ICU	Tunjuelito
CO_R282	F	70	Urine	None	Dead	Room	San Cristobal
CO_R425	M	70	Urine	None	Dead	ICU	Kennedy
CO_B41	M	44	BAL	Fluconazole	Alive	Room	Chapinero
CO_B44	F	26	Vaginal swab	N.D	N.D	N.D.	Chapinero
CO_B69	F	68	Faeces	None	Alive	Room	Chapinero
CO_B60	M	94	Urine	None	Alive	Room	Chapinero
CO_B77	M	18	BAL	None	Alive	Room	Chapinero
CO_B80	F	73	BAL	Voriconazole, caspofungin, amphotericin B	Dead	Room	Chapinero

*BAL* bronchoalveolar lavage, *N.D* No data

The nosocomial isolates reported here will be deposited at the Belgian Coordinated Collections of Micro-organisms (BCCM/MUCL) once national and international regulations regarding biological material transport have been met.

The *C. albicans* SC5314 clinical reference strain, *C. albicans* ATCC 90028, *C. africana* ATCC 2669 and *C. dubliniensis* ATCC MYA-646 reference strains were tested in conventional identification assays as controls. The *Candida glabrata* ATCC 2001 reference strain was used as negative control in chlamydospore formation assays.

### Culture medium

Each isolate was cultured using identical conditions. The samples were inoculated into Sabouraud agar (Difco, St Louis, Mo) for 24–48 h at 30, 37, 42 and 45 °C. Identity was confirmed by conventional identification methods, such as germ tube induction at 37 °C, microscopic morphology and chlamydospore formation in corn meal agar (Oxoid, Basingstoke, United Kingdom) [23]. The SC5314 strain was used as positive control for chlamydospore formation and the *Candida glabrata* ATCC 2001 strain as negative control. Photographs were taken with an optical light microscope (Leica icc50HD) at 40×.

Samples were also plated on CHROMagar (Becton Dickson, Meylan, France) to verify the clinical isolates’ chromogenic presentation, read after 48 h incubation, according to the manufacturer’s recommendations. The carbohydrate assimilation pattern was evaluated in standardised serial assays on yeast nitrogen base (YNB) agar, containing trehalose, glucosamine and then sucrose as sole carbon source (5 g/L). The amount of yeast inoculated into the medium was 10^3^ CFU; this was read after 2 and 5 days incubation at 30 °C [5].

### Isolate identification using API 20C AUX

Fresh cells were collected after culturing in Sabouraud medium for 48 h at 30 °C; an API 20 C AUX (bioMériux, France) kit was then used, following the manufacturer’s recommendations. Growth in each cupule was visually inspected after 48 h. The codes so obtained were analysed using apiweb software (bioMériux, France).

### Amplifying and sequencing molecular markers

The D1/D2 region of the rRNA gene complex 28 subunit was amplified following international guidelines for the molecular identification of fungi for *Candida* identification [10, 12]. The hyphal cell wall protein 1 (*HWP1*) gene [6] was also used. Genomic DNA was extracted from isolated colonies grown in Sabouraud dextrose agar (Becton Dickinson and Co.) using a QIAamp DNA mini kit (QIAGEN, Germantown, MD), following the manufacturer’s recommendations. The molecular markers were amplified using the primers and protocols previously described for the D1/D2 region [12] and the *HWP1* gene [6].

The amplified products from the D1/D2 region (~600 bp) were sent to Macrogen (Maryland, USA) for Sanger bidirectional sequencing. Sequencher 5.0 software (Gene Code Corporation) was used for editing and aligning the sequences. A search was then made in the following databases for each sequence to establish similarity with known strains: the NCBI databases (BLAST) (National Center for Biotechnology Information, Washington, DC), CBS-KNAW (Fungal Biodiversity Centre) and Mycobank database (International Mycological Association).

Typing by *HWP1* gene was based on the amplified products’ difference in size: ~ 700 bp for *C. africana*, ~ 941 bp for *C. albicans* and ~ 569 bp for *C. dubliniensis* [6]. The assays were done in triplicate; amplification product size was assessed on agarose gels.

### MALDI-TOF MS

The clinical isolates were cultured in Sabouraud agar for 24–48 h at 30 °C. MALDI-TOF MS was used for identification, using the protein extraction in formic acid/ethanol method, according to the Bruker Daltonics’ protocol with minor modifications as reported by Cendejas-Bueno et al., in 2012 [24]. The identification spectrum was produced from 240 laser shots in duplicate and compared to the equipment’s (Bruker) mass spectrum library. Twenty measurements were made for each clinical isolate. The MALDI-TOF MS results were then compared and a score was obtained according to the manufacturer’s technical specifications as follows: correct genus and species identification (≥2.0), secure genus identification (1.7–2.0) and no reliable identification (<1.7).

MS identification during measurements was visualised by Bruker flex analysis software and MALDI Biotyper RTC [24]. The original, commercially-available Bruker database, BDAL, is regularly updated by the manufacturer. This research was carried out with a BDAL library containing 4110 main spectra (MSPs) created between 2007 and 2012.

### Maldi spectra analysis

Peak readings having less than 5 % relative intensity and signal/noise less than 3 were eliminated. The spectra obtained from each sample (isolates and reference strains) were combined, resulting in 15 spectra (11 from the clinical isolates, two from *C. albicans* (strains 90028, and SC5314), one from *C. africana* (strain ATCC 2699) and one from *C. dubliniensis* (strain ATCC MYA-646).

Data binning for each spectrum reduced the effects of minor observation errors, using 10 m/z as bin interval (ranging from 2000 to 15,380). The presence of a peak in a bin was scored as 1, its absence as 0. Distance matrices were estimated on the resulting binary matrix (15 × 559) using absolute Pearson and Euclidian measures; a cluster analysis was then performed using UPGMA and complete methods. Unbiased bootstrap values were used to support the obtained trees (1000 replicas), all using the R 3.1.3 *pvclust* package [25–27]. Metric multi-dimensional scaling (MDS) was also performed on each distance matrix, using the R 3.1.3 *stats* package [26].

## Results

### Clinical features characterising the collected samples

The samples were collected in six third-level hospitals in Bogotá, Colombia. Samples labelled CO_B were isolated from clinical settings involving one teaching hospital (San Ignacio); those labelled CO_R came from 5 third-level hospitals, most of them (4/5) being isolated from intensive care units (ICU). The clinical information related to the samples revealed that only one of them involved vaginal flow; the remainder came from urine, bronchoalveolar lavage or blood. There were no significant differences regarding the 11 patients’ gender and half of them were more than 60 years-old. Four of them had received antifungal treatment associated with a diagnosis of *Candida* infection. Table 1 shows that patients received one or several of the following antifungals in the doses shown according to each patient’s clinical condition (i.e., urinary tract infection, invasive candidiasis and candidaemia): fluconazole (400–800 mg/day), voriconazole (6/3/mg/kg/day), caspofungin (70/50 mg) or amphotericin B (0.7–1.0 mg/kg), following international protocols [28]. Even though this was a small sample, it should be stated that four out of this group of 11 patients died and one of these patients died after having received sequential treatment involving voriconazole, caspofungin and amphotericin B due to invasive candidiasis. All patients had an underlying condition; this was cancer-related in two cases, one had hepatic failure and another one suffered chronic renal failure (Table 1).

### Identifying atypical *Candida albicans* by MALDI-TOF MS

Identification consisted of two phases; the first involved a batch of 40 nosocomial samples (CO_R) from third-level hospitals in Bogotá which were sent to CBS-KNAW in Utrecht, the Netherlands. MALDI-TOF MS output classified 5 of them as *C. albicans-C. africana*. These 5 atypical ones were then confirmed in Colombia in duplicate. Two hundred and forty samples were obtained from the San Ignacio teaching hospital (CO_B) and processed during a second phase by MALDI-TOF MS, an extra 6 isolates being classified as *C. albicans-C. africana*, following protein extraction in formic acid/ethanol by the Universidad Javeriana’s Human Proteomics and Mycosis Research Unit (Bogotá, Colombia).

MALDI-TOF MS provided identification with >2.0 scores for all strains tested in duplicate; analysis included the *Candida albicans* ATCC 90028, SC5314, *C. africana* ATCC 2669 and *Candida dubliniensis* ATCC MYA-646 reference strains and the clinical isolates (Table 2).

**Table 2:** Clinical isolates’ phenotypical and genotypic characterisation

	*Candida albicans*	*Candida dubliniensis*	*Atypical Candida albicans*										
	SC5314	MYA-646	CO_B41	CO_B44	CO_B60	CO_B69	CO_B77	CO_B80	CO_R6	CO_R41	CO_R111	CO_R282	CO_R425
Morphology													
Germ tube formation	+	+	+	+	+	+	+	+	+	+	+	+	+
Chlamydospore production	+	+	-	-	-	-	-	-	-	-	-	-	-
Pseudohyphae	+	+	+/−	+/−	+/−	+/−	+/−	+/−	+/−	+/−	+/−	+/−	+/−
Substrate assimilation													
Trehalose	+	+	-	-	-	-	-	-	-	-	-	-	-
Glucosamine	+	+	-	-	-	-	-	-	-	-	-	-	-
Sucrose	+	+	+	+	+	+	+	+	+	+	+	+	+
API 20C AUX Code	2576174	6152034	2576034	2576034	2576034	2576034	2576034	2576034	2576034	2576034	2576034	2576034	2576034
Growth													
At 30 °C	+	+	+	+	+	+	+	+	+	+	+	+	+
At 37 °C	+	+	+	+	+	+	+	+	+	+	+	+	+
At 42 °C	+	-	-	-	-	-	-	-	-	-	-	-	-
At 45 °C	+	-	-	-	-	-	-	-	-	-	-	-	-
Growth in chromogenic medium													
CHROMagar	Green	Green	Green	Green	Green	Green	Green	Green	Green	Green	Green	Green	Green
Molecular markers													
D1/D2 domains			100 % identity with *C. albicans*	100 % identity with *C. albicans*	100 % identity with *C. albicans*	100 % identity with *C. albicans*	100 % identity with *C. albicans*	100 % identity with *C. albicans*	99 % identity with *C. albicans*	100 % identity with *C. albicans*	100 % identity with *C. albicans*	100 % identity with *C. albicans*	100 % identity with *C. albicans*
*HWP1* gene	940 bp	569 bp	940 bp	940 bp	940 bp	940 bp	940 bp	940 bp	940 bp	940 bp	940 bp	940 bp	940 bp
Bruker MALDI Biotyper Library													
Score > 2.0	*C. albicans*	*C. dubliniensis*	*C. albicans -africana*	*C. albicans -africana*	*C. albicans -africana*	*C. albicans -africana*	*C. albicans -africana*	*C. albicans -africana*	*C. albicans -africana*	*C. albicans -africana*	*C. albicans -africana*	*C. albicans -africana*	*C. albicans -africana*

### Identifying atypical *Candida albicans* by morphological, physiological and molecular markers

**Fig. 1:**
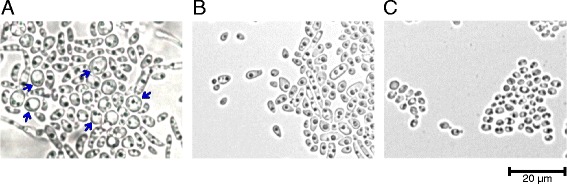
Evaluating chlamydospore production in corn meal agar. **a**. *Candida albicans* SC5314 (positive control): chlamydospores were observed after 5 days incubation (blue arrows). **b**. CO_R6 clinical isolate: no chlamydospores were observed after the same incubation period. **c**. *Candida glabrata* ATCC 2001 (negative control): no chlamydospores observed. Image captured by Motic BA200 light microscope (40×)

As MALDI-TOF MS revealed atypical *C. albicans* isolates, this led to conventional fungal identification tests. One such test involved growth in corn meal agar for verifying chlamydospore formation. Chlamydospore formation was not observed in any of the 11 atypical isolates analysed after a 5-day incubation (Fig. 1b). Three biological repeats were performed. The SC5314 strain was used as positive control, revealing chlamydospore formation (Fig. 1a), and *Candida glabrata* ATCC 2001 as negative control which showed no chlamydospore formation (Fig. 1c).

Regarding carbon assimilation tests, it was found that the 11 clinical isolates did not assimilate trehalose or glucosamine in culture medium; moreover, API 20C AUX test results showed that these isolates did not assimilate N-acetyl-glucosamine (like the *C. africana* ATCC 2669 reference strain). By contrast, the *C. albicans* SC5314 and ATCC 90028 control strains were able to assimilate trehalose, glucosamine and N-acetyl-glucosamine. All clinical isolates were unable to grow at 42 °C in growth temperature tests. Table 2 summarises the main findings, comparing the clinical *C. albicans* reference strain SC5314 and *C. dubliniensis* ATCC MYA-646 (as a related strain) to atypical isolates.

Concerning molecular markers, D1/D2 sequences from the 11 isolates had ≥99 % sequence identity with *C. albicans* (Table 2). Regarding the *HWP1* gene amplification, all clinical isolates had the ~940 bp band characteristic of *C. albicans*, whilst the *C. africana* ATCC 2669 had the expected ~ 700 bp amplicon and *C. dubliniensis* control strain ATCC MYA-646 displayed the expected ~569 bp amplicon.

### Cluster analysis and metric multidimensional scaling (MDS)

**Fig. 2:**
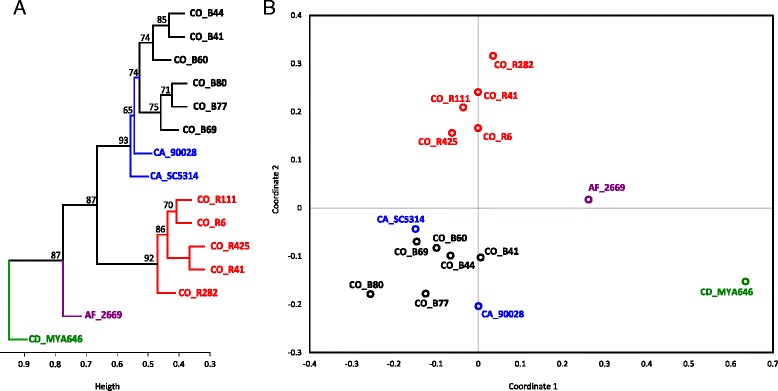
**a**. Cluster analysis. The dendrogram was calculated using UPGMA on an absolute correlation distance matrix. Weighted bootstrap values are indicated (>95 is considered well supported). **b**. Metric multi-dimensional scaling (MDS). The scatter plot shows the 2D projection of the absolute correlation matrix. Mass signatures of reference spectra (r) from *C. albicans* (strains ATCC 90028, SC5314, in blue), *C. dubliniensis* (ATCC MYA-646 in green) and *C. africana* (ATCC 2669 in purple). Clinical isolates from San Ignacio hospital: CO_B (*in black*). Clinical isolates from other hospitals: CO_R (*in red*)

Cluster analysis (Fig. 2a) and MDS (Fig. 2b), regardless of the clustering method and distance used, showed four well supported groups. *C. dubliniensis* (green) reference strain was clustered as a differentiated element as well as *C. africana* (purple) reference strain. The clinical isolates coded CO_B (black) from the San Ignacio hospital were associated with the *C. albicans* strains. The CO_R isolates (red), collected from five third-level hospitals different to San Ignacio, were grouped in an independent group, but related to the CO_B-*C. albicans* group.

## Discussion

This study highlights the usefulness of combining molecular and phenotypical traits for identifying *Candida* nosocomial isolates. MALDI-TOF MS was used as the first screening method, reporting the 11 clinical isolates as *C. albicans* / *C. africana*. This technique has been validated as a methodology which improves the diagnosis of fungal infection, having 98.5 % correct identification for *C. albicans* when the protein extraction protocol is followed by identification involving spectrometry [13, 29].

Clinical isolates were identified differently when molecular markers were used; all clinical isolates were *C. albicans* according to D1/D2 sequencing. However, several authors have stated that the small difference in sequence (just 1 %) between *C. albicans* and *C. africana* using this molecular marker does not properly differentiate between these two species [7, 30]. The *HWP1* gene was thus used to overcome this problem as it has been reported as being appropriate for differentiating *C. albicans, C. africana* and *C. dubliniensis* [6]. This assay, when performed according to Romeo et al., 2008 [6], identified our 11 clinical atypical isolates as *C. albicans* (Table 2)*.* A similar result has been described in a report of a clinical case in Italy where the amplification product obtained was equal in size to that found for the *C. albicans* reference strain, but different to that from *C. dubliniensis*, its closely-related species, even though other findings using conventional identification agreed with *C. africana* typing [31]. Such findings supported Romeo and Criseo’s notion that *HWP1* is a useful marker; however, additional methodologies are needed for atypical species to differentiate the species belonging to this complex [16].

A third line of evidence was established using chlamydospore formation as a morphological trait differentiating *C. albicans* (+ for chlamydospore formation) from *C. africana* (—for chlamydospore formation) (Table 2). This criterion has been shown to have physiological relevance since chlamydospore-forming species tend to resist stress conditions [5]. The remaining phenotypical criteria regarding our clinical isolates also agreed with typical *C. africana* characteristics, such as an inability to assimilate trehalose, glucosamine or N-acetyl-glucosamine [5].

According to Tietz et al., [5] these morphological and physiological characteristics are very important taxonomic criteria which should have led to identifying our atypical isolates as *C. africana* morphospecies; by contrast, molecular markers would have indicated that these isolates belonged to *C. albicans*.

Such findings question the taxonomic position of these fungi. Some authors consider *C. albicans* as a species complex consisting of related species, such as *C. africana, C. stellatoidea* and minor species such as *C. dubliniensis*. Some clinical particularities are always associated with a species, i.e., vaginal infection regarding *C. africana* and a preceding compromise involving HIV and oral localisation regarding *C. dubliniensis,* seeming to be more prevalent in cystic fibrosis patients [9, 19, 32]. *C. africana* infection has been described worldwide in Senegal, Madagascar, Nigeria, Angola, Germany, Italy, the UK, Spain, Saudi Arabia, India, Japan, the USA, Chile and China [7, 11, 19, 33–37]. The authors of such reports have highlighted the epidemiological importance of correlating these species’ presence with their true impact on human pathology.

The current work has been the first to describe nosocomial isolates identified as atypical *C. albicans* as their morphological and substrate assimilation fit with *C. africana.* Concerning the clinical data reported here, only one isolate came from vaginal infection whilst the remaining isolates came from urine, blood, faeces or bronchoalveolar lavage, meaning that the infection spectrum was broader and was even associated with triggering the death of some patients (4/11). However, underlying conditions pertaining to comorbidity have also to be taken into account. The mortality rate could not be accurately attributed due to the small sample size, thereby indicating the need for further studies involving an active search for cases to better establish these atypical species’ degree of impact [19].

Several authors have mentioned new challenges arising from finding cryptic *Candida* species, as well as the available methodology for precisely differentiating variants which do not fit conventional identification parameters [16]. MALDI-TOF MS is a useful tool for improving fungal disease diagnosis [29, 38]. MALDI-TOF MS spectra were directly analysed here as an additional source of information, in a similar way to that reported by Qian et al. [25].

Clustering and MDS results revealed well-defined differences amongst *C. albicans*, *C. africana* and *C. dubliniensis* reference strains and, more interestingly, two groups of clinical isolates, one formed by CO_B isolates, clearly related to *C. albicans* reference strains, and another formed by CO_R samples. Two different forces modelling clinical isolates’ clustering pattern may thus be proposed. Place of origin and an ICU background (related to prophylactic antifungal therapy) can provide these pathogens’ local epidemiological setting. CO_R isolates came from different hospitals, but had a common background related to critical patients’ conditions; 4 of the 5 were hospitalised in an ICU and 3 of the 5 in this group died. Previous exposure to fluconazole could be assumed in these nosocomial isolates, according to ICU protocols or these patients’ critical condition (Table 1). Otherwise, the same place of origin was a common characteristic for CO_B isolates from a clinical setting involving only one hospital; these isolates came from patients who had not been in an ICU, so this group of patients did not have a critical condition related to prophylactic antifungal therapy. Consequently, the grouping observed for the isolates from both groups might have reflected differential selective drug pressure and origin.

MALDI-TOF MS spectra analysis may improve information regarding each hospital’s local epidemiology; this could be used as an effective tool for infection control since the information obtained through MALDI spectra allows building local libraries closely representing the real nosocomial flora in each hospital [39]. This highlights the challenge of establishing preventative control in emerging resistance to antifungal therapy.

The foregoing identifies one of the key aspects in defining diagnosis and treatment, namely the need for timely, accurate methodologies for identifying microorganisms, thus facilitating decision-making regarding appropriate treatment and hospital infection control surveillance programmes. What happens regarding nosocomial flora would thus be clearly defined when decisions need to be made regarding epidemiological control and/or studying outbreaks of disease [2].

## Conclusions

Our results have highlighted the need for additional markers (both phenotypical and molecular) for resolving clinical isolates’ identity. The usefulness of combining MALDI-TOF MS with molecular, morphological and physiological approaches in identifying atypical *Candida albicans* isolates has been shown.

The samples analysed here were classified as *C. albicans* by the *HWP1* molecular marker which is useful in differentiating between *C. albicans*-related and minor species. Samples also had characteristics compatible with *C. africana*, as assessed by conventional mycology methods, such as the absence of chlamydospore formation, an inability to assimilate trehalose, glucosamine or N-acetyl-glucosamine and inability to grow at 42 °C.

Classification based on clinical isolates’ spectra, comparing them to reference strain spectra, led to interesting findings regarding the local epidemiology of the hospitals participating in this study. This highlighted such statistical strategies’ usefulness as a tool for approaching problems concerned with nosocomial infection involving atypical germs. The reference *C. albicans*-related and minor species used were also successfully differentiated; moreover CO_B and *C. albicans* constituted a common cluster, whilst CO_R constituted a well differentiated cluster, meaning that it could be a different bio-variant of *Candida*, but still related to *C. albicans*. These isolates could thus be considered atypical *C. albicans.*

Evidence has been presented here concerning the importance of a prophylactic antifungal therapy-related pharmacological background which can be used as a selective force determining the clustering of a specific isolate (CO_R) that reflects common practice in ICUs in the different hospitals sampled. This would agree with findings by Sydnor et al.*,* (2011) [2] regarding the specific hospital location of nosocomial flora and a relationship with and maybe a common origin for ICU *Candida* isolates in the samples reported here from different hospitals.

CO_B isolates had a common hospital origin and more diverse clinical settings, lacking antifungal prophylactic guidelines. This factor could lead to less stressful conditions for nosocomial isolates and less selective pressure; the latter has been considered by Maubon et al., (2014) [40] as being one of the challenges in establishing preventative control of emerging resistance to antifungal therapy. These results suggest that more studies should be carried out to evaluate whether different antifungal susceptibility exists between these groups.

The clinical and epidemiological aspects of infection are relevant, since the pathological implications of these atypical *C. albicans* isolates require further molecular and susceptibility studies for understanding their ability to adapt (this includes phenotypic plasticity) and their potential for harming a vulnerable host.

## Abbreviations

BAL: Bronchoalveolar lavage
HIV: Human immunodeficiency virus
MDS: Multi-dimensional scaling
UPGMA: Unweighted pair group method with arithmetic mean
MALDI-TOF MS: Matrix-assisted laser desorption/ionisation time of flight mass spectrometry
